# Facilitate Insight by Non-Invasive Brain Stimulation

**DOI:** 10.1371/journal.pone.0016655

**Published:** 2011-02-02

**Authors:** Richard P. Chi, Allan W. Snyder

**Affiliations:** Centre for the Mind, University of Sydney, Sydney, Australia; University of Oxford, United Kingdom

## Abstract

Our experiences can blind us. Once we have learned to solve problems by one method, we often have difficulties in generating solutions involving a different kind of insight. Yet there is evidence that people with brain lesions are sometimes more resistant to this so-called mental set effect. This inspired us to investigate whether the mental set effect can be reduced by non-invasive brain stimulation. 60 healthy right-handed participants were asked to take an insight problem solving task while receiving transcranial direct current stimulation (tDCS) to the anterior temporal lobes (ATL). Only 20% of participants solved an insight problem with sham stimulation (control), whereas 3 times as many participants did so (p = 0.011) with cathodal stimulation (decreased excitability) of the left ATL together with anodal stimulation (increased excitability) of the right ATL. We found hemispheric differences in that a stimulation montage involving the opposite polarities did not facilitate performance. Our findings are consistent with the theory that inhibition to the left ATL can lead to a cognitive style that is less influenced by mental templates and that the right ATL may be associated with insight or novel meaning. Further studies including neurophysiological imaging are needed to elucidate the specific mechanisms leading to the enhancement.

## Introduction

Thinking outside the box is difficult. And counter-intuitively, those with the most in-depth knowledge do not have an advantage in this pursuit [1]. In fact, as Kuhn [2] noted, “almost always the men who achieve these fundamental inventions have been either very young or very new to the field whose paradigm they change.” One possible explanation for this paradox is that our mind is hypothesis driven [3], [4]. In other words, our observations of the world are strongly shaped by our preconceptions. For example, information consistent with our expectations or mental templates is often accepted at face value, whereas inconsistent evidence is discounted or hidden from conscious awareness [5]. While this hypothesis driven mechanism helps us in efficiently dealing with the familiar, it can prevent us from seeing better solutions in a different and/or unfamiliar context [6].

Presumably, it would be beneficial in certain situations if we could *temporarily* induce a state of mind that is less top-down, in other words, less influenced by mental templates or preconceptions. Interestingly, a clue for achieving this comes from people with brain dysfunctions [7], [8]. For example, Miller et al. [9] found that artistic talent, due to a different way of perceiving the world, can sometimes emerge spontaneously in those with dominant (usually left) anterior temporal lobe dementia. They argued that damage to this area may interrupt certain inhibitory mechanisms in the left hemisphere and disinhibit contralateral areas in the right. As an oversimplified caricature, brain dysfunctions, induced or caused by inhibiting and disinhibiting certain neural networks, may make our cognitive style less hypothesis driven, thereby enabling access to a level of perception normally hidden from conscious awareness [7], [8].

This raises a provocative possibility: Can we facilitate insight problem solving in healthy people by *temporarily* inhibiting or disinhibiting certain areas of the brain? To explore this possibility, we used transcranial direct current stimulation (tDCS) (see Methods), a safe, non-invasive technique that can increase or decrease cortical excitability and spontaneous neuronal firing in the stimulated region depending on current polarity [10], [11].

We hypothesized that cathodal stimulation (decreasing excitability) of the left anterior temporal lobe (ATL) together with anodal stimulation (increasing excitability) of the right ATL would facilitate performance on an insight problem solving task. This prediction is based on evidence that the right ATL is an area associated with insight [12], [13] and novel meaning [14] and that inhibition of the left ATL is associated with emergence of certain cognitive skills and a less top-down or hypothesis driven cognitive style [9], [15], [16], [17]. More generally, it is consistent with evidence that the left hemisphere is involved in the maintenance of existing hypotheses and representations [18], [19], [20], [21], while the right hemisphere is associated with novelty and with updating hypotheses and representations [22], [23], [24], [25], [26]. We elaborate further on this in the Discussion.

## Methods

### Participants

67 healthy right handed subjects aged between 18 and 38 years from the University of Sydney participated in our study, with 60 participants included in the final analysis. Individuals with a score greater than 50 on the Edinburgh Handedness Inventory [27] were eligible for participation. Participants were screened and excluded if they had any neuropsychiatric disorder, current or past history of drug use, were taking any medication acting on the central nervous system or were pregnant.

Of the 67 participants, 5 participants who had previous experience with the task (matchsticks arithmetic problems) were excluded. 2 other participants who had abnormal difficulties with Roman numerals and/or learning our testing protocols were also excluded. Therefore, after exclusion, data from sixty participants (29 females, mean age = 22, SD = 3.9) were used in this study (See Table 1 for demographic characteristics across the three stimulation groups). All of these participants were naïve to tDCS and had no prior experience with the matchstick insight problem solving task. The study was carried out to conform to the principles of the Declaration of Helsinki and was approved by the University of Sydney Human Research Ethics Committee. All participants gave written informed consent for the study prior to the experiment.

**Table 1:** Demographic characteristics across the three stimulation groups.

	Sham	L− R+	L+ R−
Age (years)	21.9±0.72	23.8±1.1	21.8±0.63
Gender (number of females)	14	5	12
Time required in completing the mental set phase (seconds)	536±186	555±128	442±99
Experience in a quantitative field (number of participants)			
Limited	4	6	4
Average	8	9	10
Significant	8	5	6

Values are presented as mean ± standard error of the mean. Participants across the three stimulation groups did not differ in terms of age (p = 0.19, ANOVA), time required in completing the mental set phase (p = 0.76, ANOVA) or experience in a quantitative field (p = 0.85, 2 tailed Fisher's exact test). It turned out that gender is not evenly distributed across the stimulation groups, with a few more females in the sham stimulation group. Nevertheless, it is clear from the data that gender is not a predictor of success in problem solving for either the Type 2 (p = 1, 2-tailed Fisher's exact test) or Type 3 (p = 0.58, 2-tailed Fisher's exact test) insight problem (see Table 2).

### Transcranial direct current stimulation (tDCS)

tDCS involves applying a weak direct current to the scalp via two saline-soaked sponge electrodes, thereby polarizing the underlying brain tissue with electrical fields. It has been shown that tDCS can modulate cortical excitability and spontaneous firing activities in the stimulated region by shifting the resting membrane potential [28]. Depending on the polarity of the current flow, cortical excitability can be increased (anodal stimulation) or decreased (cathodal stimulation) during and beyond the period of stimulation [10], [29]. It is an ideal neuromodulation technique for our purpose because it is safe and has a particularly effective placebo that blinds subjects from stimulation conditions [30].

We used a custom made, battery-driven, constant current stimulator with a maximum output of 2mA and 2 sponge electrodes each with an area of 35cm2. Our device is particularly reliable for blinding subjects to stimulation conditions because it can be set to an ON display even when there is no stimulation (as in the sham, or control, condition).

For the active stimulation conditions, a constant current of 1.6mA intensity was applied, and was manually and slowly ramped up and down (over 30 seconds). The current density is 1.6mA/35cm2 which is equal to 0.0457mA/cm2. For the sham stimulation (control) condition, the sponge electrodes were placed in the same positions as in active stimulation, but after 30 seconds, the electrical current was covertly ramped down so that subjects did not receive further stimulation for the rest of the experiment. Gandiga et al. [30] suggested that the “sham stimulation” described above can blind subjects from stimulation conditions since this method produces similar initial tingling sensations in subjects as active stimulation does. In addition, to ensure that the blinding would be successful, we chose 1.6mA instead of 2mA as the intensity for the active conditions. This was based on previous experiences with tDCS, in which we noted that some participants felt particularly noticeable tingling sensations when the intensity was increased above 1.6mA.

We used a between-subjects design in accordance to Ollinger el al. [31], rather than a repeated measure design, to prevent practice effects from cofounding our results.

The 60 right handed participants were randomly assigned to one of three types of stimulation prior to the start of the experiment: 1) Cathodal stimulation of the left ATL together with anodal stimulation of the right ATL. This is referred in the text as the “L− R+ stimulation” condition. Specifically, the cathode electrode was placed over at the left ATL, approximately half way between T7 and FT7 on the International 10–20 System for electrode placement. The anodal electrode was placed over at the right ATL, approximately half way between T8 and FT8 on the same 10–20 System. The area is laterally 40% of the intra-auricular distance from the vertex and anteriorly 5% of the distance from inion to nasion. The areas were determined with the guidance of an EEG cap. 2) Anodal stimulation of the left ATL together with cathodal stimulation of the right ATL. This is referred to as the “L+ R− stimulation”. 3) “Sham stimulation” for control, involving transient, non-effective stimulation in the L− R+ configuration (i.e. the same placement as in condition 1). Participants were blind to their stimulation condition.

None of the participants experienced adverse effects as a result of tDCS or withdrew from the study.

### Cognitive task

**Figure 1:**
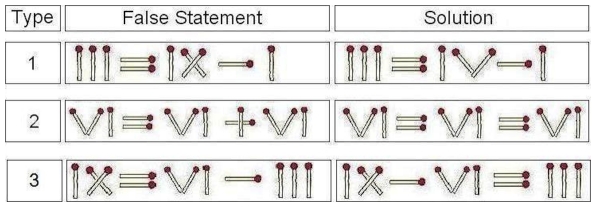
An illustration of the insight problems used. Type 1 insight problems were used in the mental set phase. Type 2 and Type 3 problems were used in the testing phase.

To assess whether we could facilitate insight, we used a well known experimental paradigm involving “matchstick arithmetic” [31]. Participants were asked to correct a false arithmetic statement, presented in Roman numerals constructed from matchsticks, by moving one stick from one position to another position without adding or discarding a stick (see figure 1). The only valid symbols were the Roman numerals ‘I’, ‘V’, ‘X’ and the arithemetic operators ‘+’, ‘−’ and ‘ = ’. We followed the procedure of Ollinger et al. [31] who demonstrated that repeatedly solving problems requiring one kind of insight (e.g. changing an X to a V as shown in Type 1 of figure 1) impairs subsequent performance on problems requiring a different kind of insight (e.g. changing a + sign to an  =  sign as shown in Type 2 of figure 1). In fact, they found that only 10% of participants could solve the Type 2 problem shown in figure 1 after solving a series of 27 Type 1 problems [31].

### Procedure

The experiment was conducted in a quiet room with no distractions. Participants were told that we were investigating the effect of brain stimulation on a matchstick problem solving task. They were first given computerised instructions for the matchstick task and a practice task of 3 Type 1 Problems (e.g. see figure 1) composed of actual matchsticks on the table in front of them. The experimenter demonstrated the correct solution if the participant could not solve any practice item. Throughout the experiment, participants were given a Roman numeral table from 1 to 15 and actual matchsticks that they could use to help them reach the solution.

During the mental set phase, participants were asked to solve a series of 27 Type 1 problems presented one at a time via Microsoft PowerPoint. The solutions for all of these problems involve changing an ‘X’ to a ‘V’ by moving a stick. Participants had up to 2 minutes per problem and were asked to report the solution out loud when they found it. They were given the solution during this mental set phase if they could not solve the problem after 2 minutes.

After the mental set phase, participants were told that they would receive 5 minutes of tDCS before being asked to solve a few additional problems. They were also told that the stimulation would continue until the end of the second (testing) phase. tDCS was initiated *after* the mental set phase (solving the 27 Type 1 problems) and 5 minutes *before* initiating the testing phase because cortical excitability changes induced by tDCS are not usually observed until after a period of 3–5 minutes [10].

After the 5 minutes of tDCS, participants began the testing phase when they were asked to solve 2 additional problems (the Type 2 and Type 3 problems as shown in figure 1). During the testing phase, participants were given up to 6 minutes for each of the 2 test problems (the order of presentation was counterbalanced) and were not given the correct solutions if they failed. Stimulation *continued* until the end of the testing phase (up to a maximum of 17 minutes).

### Statistical analysis

The primary dependent variable was the number of subjects who could solve the most difficult insight problem (Type 2) during the testing phase by the end of 360 seconds. We specifically focused on results for the harder (Type 2) insight problem because brain lesions have been shown to produce an advantage only for these problems, not for the easier (Type 3) problems [32]. However, to replicate the experimental procedure of Ollinger et al. [31], we also undertook an exploratory analysis of the results for the Type 3 problem.

A two-tailed Fisher's exact test was used to test the prediction that those in the L− R+ stimulation group would have a higher success rate in solving the insight problems than those in the sham stimulation group. In addition, a survival (time to event) analysis was used to compare whether there was any difference in the time to event curves between the L− R+ group and the sham stimulation group. Specifically, “event” is defined as solving the insight problem (Type 2) during the testing phase. Time to event curves (censored at 360 seconds) were plotted using the Kaplan-Meier method and comparisons between the curves were analysed using the logrank test [33].

In summary, Fisher's exact test and the logrank test were used to assess the prediction that those in the L− R+ group would perform better than those in the sham stimulation group. In contrast, we did not have a hypothesis for those in the L+ R− group, for several reasons (see Discussion), so the data for the L+ R− group were subjected to exploratory analyses.

## Results

Overall, condition of stimulation had a significant effect on the time to event curves for both the Type 2 insight problem (p = 0.010, logrank test) and the Type 3 problem (p = 0.037, logrank test). Condition of stimulation also had a significant effect on performance at the end of 6 minutes for both the Type 2 problem (p = 0.024, two-tailed Fisher's exact test) and the Type 3 problem (p = 0.034, two-tailed Fisher's exact test).

**Figure 2:**
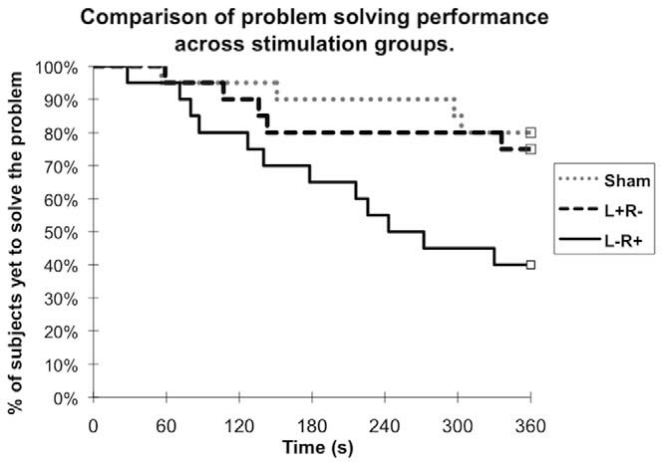
The figure provides a comparison of problem solving performance (Type 2 insight problem) across stimulation groups. Condition of stimulation has a significant effect on both the time to event (solving the Type 2 insight problem) curves (p = 0.010, logrank test) and the percentage of subjects who solved the insight problem by the end of 6 minutes (p = 0.024, 2 tail fisher's exact test). While participants in all stimulation groups had difficulties in the first minute, after 150 seconds, only those in the L− R+ group continued to solve the insight problem over time. By the end of 360 seconds, 60% of those in the L− R+ stimulation group could solve the problem whereas only 20% of those in the sham stimulation group could do so (p = 0.022, two tail fisher's exact test).

**Figure 3:**
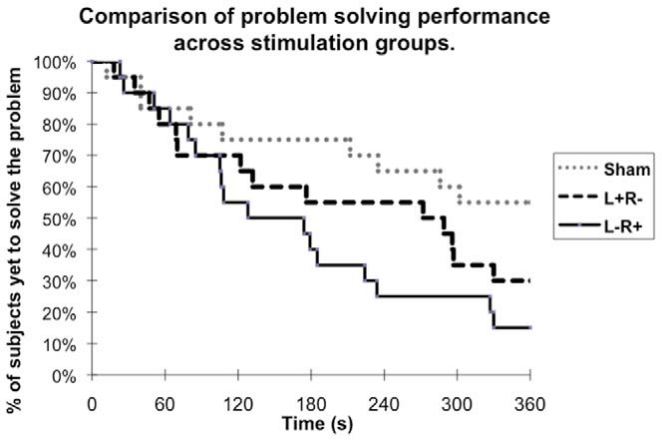
The figure provides a comparison of problem solving performance (Type 3 insight problem) across stimulation groups. We a priori did not intend to use the Type 3 insight problem to test our main hypothesis that those in the L− R+ group would perform better than those in the sham stimulation group. This is because those with brain lesion paradoxically perform better only for Type 2 problems, but not for Type 3 problems (Reveberi et al., 2007). Nevertheless, the result for the Type 3 problem is consistent with our hypothesis and also consistent with results for the Type 2 problem. Note that while the comparisons between L+ R− and sham (p = 0.15, logrank test) and between L+ R− and L− R+ (p = 0.26, logrank test) are not significant (possibly due to the lack of power), it is clear that those in the L− R+ group had a significant advantage over those in the sham stimulation group (p = 0.010, logrank test).

Our prediction, that those in the left cathodal/right anodal group (L− R+) would perform better than those in the sham group, is strongly supported by the findings (p = 0.008, logrank test) (see figure 2). Only 20% of participants in the sham stimulation (control) group solved the Type 2 (hardest) problem (shown in figure 1) by the end of 6 minutes whereas, in contrast, 60% of participants solved it in the L− R+ group (p = 0.022, two-tailed Fisher's exact test). Similarly, only 45% of participants in the sham stimulation (control) group solved the Type 3 (easier) problem (shown in figure 1) by the end of 6 minutes whereas 85% of participants who received L− R+ stimulation solved it (p = 0.019, two-tailed Fisher's exact test) (see figure 3).

Importantly, participants who received stimulation of the opposite polarity (L+ R−) did not perform differently from those in the sham group for either problem Type 2 (p = 1, 2-tailed Fisher's exact test) or Type 3 (p = 0.20, 2-tailed Fisher's exact test) at the end of six minutes. Similarly, there was no significant difference in the time to event curves between the L+ R− group and the sham stimulation group for either the Type 2 (p = 0.68, logrank test) or the Type 3 (p = 0.15, logrank test) insight problem.

Of the 60 participants included in the analysis, 57 of them solved all 27 problems in the mental set phase successfully, suggesting that most had gained proficiency in Type 1 insight problems. The 3 participants who could not solve 1 or 2 problems out of 27 Type 1 problems in the mental set phase were given the solution to these problems after 2 minutes.

There is no evidence that the 3 groups of participants differ in their problem solving abilities before tDCS (see Table 1) and most of them, regardless of stimulation condition, had difficulties in the first minute of the testing phase (see Figure 2). Furthermore, it turned out that baseline characteristics were not predictors of successful problem solving. In other words, those who solved the Type 2 or Type 3 problem did not differ from those who could not in age, gender, or experience in a quantitative field (See Table 2).

It might seem reasonable to suppose that faster performance in the mental set phase might be associated with greater (or lesser) success in the testing phase. For example, those who are faster could either be better problem solvers in general or, conversely, more stuck in the mental set. However, it turned that there was no evidence (p = 0.36, 2-tailed independent samples t test) that those who successfully solved the insight problems during the testing phase took a shorter time to complete the mental set phase.

**Table 2:** Demographic characteristics of those who were successful in solving the Type 2 problem vs those who failed.

	Success	Failure
Age (years)	21.7±0.69	22.9±0.94
Gender		
Number of females	11	19
Number of males	10	20
Time required in completing the mental set phase (seconds)	599±148	461±73
Experience in a quantitative field (number of participants)		
Limited	7	7
Average	9	18
Significant	5	14

Values are presented as mean ± standard error of the mean. Neither age (p = 0.255, 2 tailed independent t test), gender (p = 1, 2-tailed Fisher's exact test), time required in completing the mental set phase (p = 0.36, 2 tailed independent t test), or experience in a quantitative field (p = 0.36, 2-tailed Fisher's exact test) is a predictor of success in solving the Type 2 problem. In other words, there is no evidence that those in the L− R+ group had superior performance because of confounding baseline attributes.

## Discussion

The prediction that those who received L− R+ stimulation of the anterior temporal lobes would be better able to solve insight problems was strongly supported by the findings. Nevertheless, we did not expect a three-fold increase in the likelihood of solving the problems. This is the strongest cognitive enhancement we are aware of for a brain stimulation study, but we suggest that the results should be interpreted with certain limitations in mind.

Importantly, the kind of insight problem solving paradigm we used (and, arguably, any insight problem solving) involves several neural networks. Therefore, the pronounced improvement is most likely due to a combination of several mechanisms. Candidate mechanisms include diminishing a top-down (hypothesis driven) cognitive style, interrupting the mental set, improving set-switching ability, and facilitating insight directly. Even if we assume that modulation of cortical excitability by tDCS was constrained in areas strictly under the sponge electrodes (a controversial issue [34]), it is likely that this modulation would have an indirect impact on distant networks [35]. Consequently, we cannot provide a definitive explanation, and can only offer some possibilities regarding the mechanism of action leading to the enhancement we observed.

### Why tDCS improved insight?

Given our bilateral stimulation protocol, the improvement in performance could be due to decreased cortical excitability of the left hemisphere, increased excitability of the right hemisphere, or some combination of both. In any case, the model of interhemispheric rivalry [36], [37], [38], [39], which provides the rationale for many tDCS studies on stroke rehabilitation [40], predicts that both left cathodal stimulation and right anodal stimulation would have similar net effects on overall hemispheric balance. If this is true, then both the L− and R+ elements of our stimulation protocol might contribute to diminishing left hemisphere dominance, which is associated with stereotypy [20] and adherence to existing hypotheses [21], [23], [26].

This possibility is consistent with evidence that the left hemisphere is important for processing “well routinized representations and strategies” and the right hemisphere is “critical for processing novel cognitive situations” [25]. Indeed, there is evidence that those who are not strongly right handed (associated with weaker left hemisphere dominance) are more likely to update their existing mental representations [18], [21] and are less constrained by cognitive routine [24]. In other words, by diminishing left hemisphere dominance (either by L−, R+, or the combination of both), we might have increased our subjects' tendency to examine a problem anew instead of through the mental templates of well-routinized representations and strategies.

### The role of the left ATL

Alternatively, it is also possible that the pronounced improvement in insight problem solving was due solely to inhibiting (decreasing excitability of) the left ATL. This area is associated with mental templates, or context [41], [42], [43], [44] and inhibiting the left ATL can lead to a less top down influenced (hypothesis driven) cognitive style [9]. As an oversimplified caricature, by making our participants' cognitive style less hypothesis driven, less influenced by existing mental templates or context, we might have increased the chance that alternative representations, often hidden from conscious awareness (for the sake of efficiency in dealing with the familiar) are considered. Consistent with this view, Rausch [22] found that patients with left temporal lobectomy (intact right hemisphere) tended to switch hypotheses even when initial hypotheses were explicitly shown to be correct. Based on the evidence discussed above, the pronounced improvement in problem solving was possibly a result of reducing the influence of existing hypotheses, for example, reducing the impact of mental set.

### Paradoxical facilitation

Our findings are also consistent with evidence that paradoxical functional facilitation [45], such as the emergence of perceptual skills related to a less top-down cognitive style, can occur because of brain dysfunction [8], [9], [46], [47], or inhibition of the left ATL [15], [16], [17]. Consistent with this possibility, Reverberi et al. [32], using the same matchstick paradigm, demonstrated that while only 43% of healthy participants can solve the Type 2 insight problem shown in figure 1, paradoxically, 82% of patients with lesions in the lateral frontal area can do so. Such results are consistent with the view that tradeoffs or competition amongst different neural networks are common in human cognition [48], [49]. They are also consistent with the possibility that brain stimulation could modulate this tradeoff to our advantage (in certain situations) by *temporarily* inhibiting or disinhibiting certain brain regions. It would be interesting in further studies to explore whether inhibiting the lateral frontal lobe and the left ATL simultaneously by non-invasive brain stimulation would lead to an even stronger effect in improved insight problem solving.

### Increased excitability of the right ATL

Of course, it is possible that the pronounced improvement is simply due to increased excitability in the right ATL, an area associated with novel meaning [14] and insight [12], [13]. In other words, the improvement we found might be directly due to facilitating the area associated with insight rather than reducing any mental set effect. Alternatively, it is possible that tDCS can only reduce the mental set effect, but cannot facilitate insight in general. Further studies using a variety of control tasks are needed to disentangle the specific mechanisms of action and to determine whether the improvement in insight problem solving is task specific or can be widely generalized.

### Stimulation with the opposite polarity (L+ R−)

One might have anticipated (from the logic of hemispheric rivalry, discussed above) that those who received stimulation of the opposite polarity (L+ R−) would have performed worse than those in the sham condition. However, this was not the case for either problem in the testing phase. A possible explanation is that there might be a ceiling effect in that brain stimulation cannot make someone more left hemisphere dominant, more constrained by mental set, than they already are. This possibility is consistent with evidence that brain stimulation can improve the motor skills of people's non-dominant hand by decreasing excitability to the dominant motor cortex, but cannot improve people's dominant hand by increasing excitability to the dominant motor cortex [50].

Furthermore, the effect of cortical stimulation on excitability is argued to be dependent on the resting state of neurons such that stimulation might preferentially modulate less active neural networks [51]. Therefore, although cathodal stimulation, on average, will lead to decreased excitability in the stimulated region (and vice versa for anodal stimulation), it is possible that for 10–20% of the subjects, the opposite effect on cortical excitability would occur during the testing phase [51], [52]. Nevertheless, our results suggest strong hemispheric differences in that only those who received L− R+ stimulation showed an improvement. It is not the case that simply stimulating *any* brain region can improve performance by disrupting the normal state of mind.

### Limitations

As mentioned earlier, the focality of tDCS is still a controversial issue [53] and there might not be a one to one relationship between changes in cortical excitability under the electrodes and changes in brain functions [34]. On one hand, several studies modulating various brain regions have shown that the behavioural effects of tDCS are relatively focal and can lead to cognitive enhancement. For example, tDCS applied to frontal areas has been shown to improve memory [54], [55], planning [56]and complex associative thought [57], whereas tDCS applied to the parietal areas and posterior perisylvian region have led to improved visual spatial attention [36]and language acquisition [58], respectively. On the other hand, modeling studies demonstrate that there is most likely substantial current dispersion under the electrodes, especially at the cerebrospinal fluid level, where the conductance is particularly high [34], [53]. If this was the case, then the cognitive enhancement we found would be more likely a result of reducing left hemisphere dominance more globally rather than inhibiting the ATL specifically.

Furthermore, we are not able to disentangle the effect of left cathodal stimulation and right anodal stimulation in isolation to discover which has a stronger effect. We specifically used a bilateral stimulation montage with opposite polarities, which is the most efficient design for testing the primary question that tDCS can improve insight problem solving in healthy people. It also reduces the likelihood of current dispersion since unilateral stimulation (with a large monopolar electrode) by definition has a shorter distance between the electrodes and thus a higher likelihood of current shunting along the scalp [59]. Further studies might address this question with unilateral stimulation in combination with neurophysiological imaging before, during and after stimulation.

### Conclusions

Our predisposition to use contextual cues from past experience confers a clear evolutionary advantage in rapidly dealing with the familiar, but this can lead to the mental set effect or overgeneralisation. As John Maynard Keynes [60] noted, “The difficulty lies, not in the new ideas, but in escaping from the old ones, which ramify…into every corner of our mind.” Our findings suggest the possibility that brain stimulation can be used to modulate this tradeoff to our advantage in a specific situation, possibly by *temporarily* making our cognitive style less top-down influenced (hypothesis driven). For example, brain stimulation might allow a person to examine a problem anew instead of through the mental templates of what is already known. Further brain stimulation studies in combination with neurophysiological imaging and a variety of control tasks are needed to determine the specific mechanisms of actions leading to the effect and whether the pronounced cognitive enhancement we found is generalizable to other tasks.
